# Fluorine Atoms on C_6_H_5_-Corrole Affect the Interaction with M^pro^ and PL^pro^ Proteases of SARS-CoV-2: Molecular Docking and 2D-QSAR Approaches

**DOI:** 10.3390/ijms231810936

**Published:** 2022-09-19

**Authors:** Otávio Augusto Chaves, Cláudio Eduardo Rodrigues-Santos, Áurea Echevarria, Carolina Q. Sacramento, Natalia Fintelman-Rodrigues, Jairo R. Temerozo, Hugo Caire Castro-Faria-Neto, Thiago Moreno Lopes e Souza

**Affiliations:** 1 Laboratory of Immunopharmacology, Oswaldo Cruz Institute, Oswaldo Cruz Foundation (Fiocruz), Rio de Janeiro 21040-360, RJ, Brazil; 2 Center for Technological Development in Health (CDTS), National Institute for Science and Technology on Innovation on Neglected Diseases Neglected Populations (INCT/IDNP), Oswaldo Cruz Foundation (Fiocruz), Rio de Janeiro 21040-360, RJ, Brazil; 3 Department of Organic Chemistry, Institute of Chemistry, Federal Rural University of Rio de Janeiro (UFRRJ), Seropédica 23890-000, RJ, Brazil; 4 Laboratory on Thymus Research, Oswaldo Cruz Institute, Oswaldo Cruz Foundation (Fiocruz), Rio de Janeiro 21040-360, RJ, Brazil; 5 National Institute for Science and Technology on Neuroimmunomodulation (INCT/NIM), Oswaldo Cruz Institute, Oswaldo Cruz Foundation (Fiocruz), Rio de Janeiro 21040-360, RJ, Brazil

**Keywords:** corroles, SARS-CoV-2, M^pro^, PL^pro^, molecular docking, 2D-QSAR

## Abstract

The chymotrypsin-like cysteine protease (3CL^pro^, also known as main protease—M^pro^) and papain-like protease (PL^pro^) of severe acute respiratory syndrome coronavirus 2 (SARS-CoV-2) have been used as the main targets for screening potential synthetic inhibitors for posterior in vitro evaluation of the most promising compounds. In this sense, the present work reports for the first time the evaluation of the interaction between M^pro^/PL^pro^ with a series of 17 porphyrin analogues-corrole (**C1**), *meso*-aryl-corrole (**C2**), and 15 fluorinated-*meso*-aryl-corrole derivatives (**C3**–**C17**) via molecular docking calculations. The impact of fluorine atoms on *meso*-aryl-corrole structure was also evaluated in terms of binding affinity and physical-chemical properties by two-dimensional quantitative structure–activity relationship (2D-QSAR). The presence of phenyl moieties increased the binding capacity of corrole for both proteases and depending on the position of fluorine atoms might impact positively or negatively the binding capacity. For M^pro^ the *para*-fluorine atoms might decrease drastically the binding capacity, while for PL^pro^ there was a certain increase in the binding affinity of fluorinated-corroles with the increase of fluorine atoms into *meso*-aryl-corrole structure mainly from tri-fluorinated insertions. The 2D-QSAR models indicated two separated regions of higher and lower affinity for M^pro^:**C1**–**C17** based on dual electronic parameters (σ_I_ and σ_R_), as well as one model was obtained with a correlation between the docking score value of M^pro^:**C2**–**C17** and the corresponding ^13^C nuclear magnetic resonance (NMR) chemical shifts of the sp^2^ carbon atoms (δ_C-1_ and δ_C-2_) of **C2**–**C17**. Overall, the fluorinated-*meso*-aryl-corrole derivatives showed favorable in silico parameters as potential synthetic compounds for future in vitro assays on the inhibition of SARS-CoV-2 replication.

## 1. Introduction

The severe acute respiratory syndrome coronavirus 2 (SARS-CoV-2) was discovered in China (December/2019) and quickly spread globally, being classified by the World Health Organization (WHO) as a pandemic in February 2020 [1]. Since SARS-CoV-2 was discovered until the time of writing (end of August 2022), there have been over 601 million confirmed cases and 6.49 million deaths worldwide [2], overtaking both SARS-CoV and Middle East respiratory syndrome (MERS) in 2003 and 2012, respectively [3]. SARS-CoV-2 is still circulating mainly due to the variants of concern (VoC) that might escape the immune response induced by vaccines [4,5,6,7] and the few approved drugs by the U.S. Food and Drug Administration (FDA), e.g., remdesivir and baricitinib [8,9] reinforces the continuous necessity of drug development against 2019 coronavirus disease (COVID-19).

Different authors have evaluated the capacity of commercial drugs, natural products, and novel drugs to inhibit the SARS-CoV-2 proteases [10,11,12,13,14,15]. Basically, the coronaviruses have a long ribonucleic acid (RNA) strand which is used to synthesize two long polyproteins in the infected cells. The main products of these polyproteins include both structural and non-structural proteins that are responsible for the formation of new virions, and two proteases: chymotrypsin-like cysteine protease (3CL^pro^, also known as main protease-M^pro^) and papain-like protease (PL^pro^) [11,16,17,18,19]. The proteases cleavage the polyproteins into functional pieces, as example M^pro^ targets 7 and 11 cleavage sites into open reading frame (ORF) 1a and 1b, respectively, while PL^pro^ is responsible for producing non-structural protein nsp1, nsp2, and nsp3 [20,21]. The importance of M^pro^ as a target has been reinforced by the drug PF-07321332 (PAXLOVID^TM^; Pfizer), which reduced COVID-19-associated hospitalization by 80% [22,23].

Cyclic organic compounds that are based on four pyrrole moieties with three connections by methine group and only one direct pyrrole-pyrrole bond (lacking a methine group) with an extra NH proton are known as corroles (porphyrin analogues) [24]. These compounds show interesting photophysical and photochemical properties, including low aggregation tendency, strong absorption at high wavelengths, large Stokes shift, and high fluorescent quantum yield [25,26,27]. For this reason, corroles are evaluated in terms of applications in photodynamic therapy (PDT) [28], antimicrobial photodynamic therapy (aPDT) [29,30], theranostic agent [31,32], and biomacromolecules site markers [24]. Despite the fact that porphyrins have already been evaluated as potential candidates to inhibit the interaction between SARS-CoV-2 and human angiotensin-converting enzyme 2 (ACE2) [33,34], as well as photosensitizer to photodynamically destroy the SARS-CoV-2 structure [35,36], there are not any reports on the capacity of corroles to inhibit SARS-CoV-2 replication or the virus proteases.

The fluorine atom insertion into small organic compounds have been widely used in pharmacology to increase the biological properties, as an example enhance a number of pharmacokinetic and physicochemical properties such as metabolic stability, membrane permeation, and binding affinity to target proteins [37,38,39]. In the case of corroles, it has been reported that the presence of fluorine atoms may enrich the photostability, singlet oxygen production, lipophilicity, selective accumulation in tumor cells, protein binding, and catalytic antioxidants for the attenuation of diabetes mellitus [24,30].

**Figure 1:**
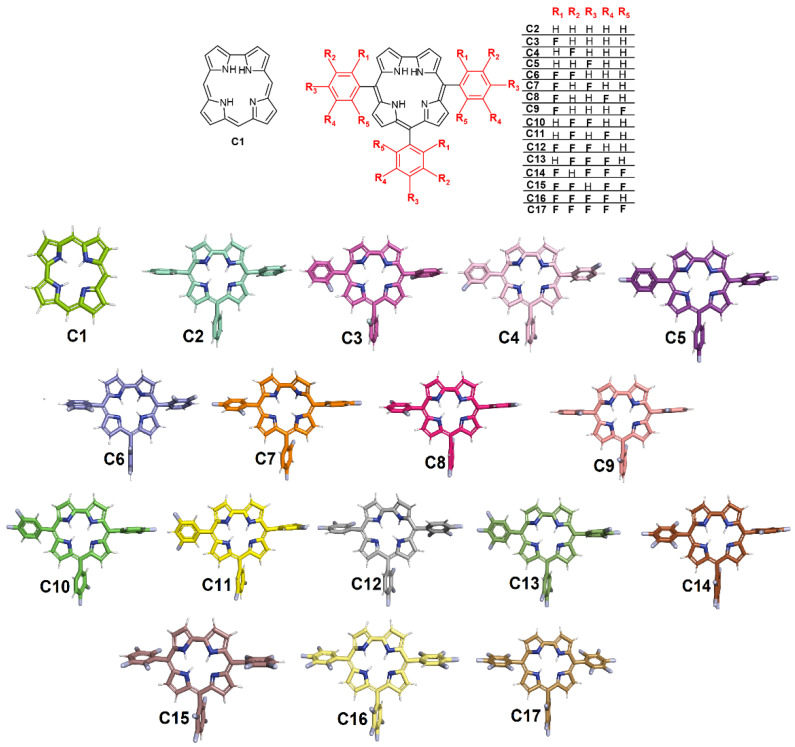
The chemical structures of corrole **C1**, *meso*-aryl-corrole (**C2**), and fluorinated-*meso*-aryl-corrole derivatives (**C3**–**C17**). The color used to represent the 3D structures of the corroles corresponding to the same used in the docking representations.

Thus, based on the high necessity of screening potential SARS-CoV-2 inhibitors, as well as the importance of corroles in the pharmacology and the positive effect of fluorine atom insertion to increase the pharmacokinetic and physicochemical properties, the present work reports the interaction between M^pro^/PL^pro^ from SARS-CoV-2 with corrole (**C1**, Figure 1), *meso*-aryl-corrole (C_6_H_5_-corrole, **C2**, Figure 1), and 15 fluorinated-*meso*-aryl-corrole derivatives (**C3**–**C17**, Figure 1) via molecular docking calculations. The impact of fluorine atoms on *meso*-aryl-corrole structure was also evaluated in terms of binding affinity and physical-chemical properties by two-dimensional quantitative structure–activity relationship (2D-QSAR). To the best of our knowledge, this is the first in silico report that explores the potential inhibitory effects of corroles against both M^pro^ and PL^pro^ proteases.

## 2. Results

### 2.1. Molecular Docking Evaluation for M^pro^ Enzyme

The Gold 2020.2 software (Cambridge Crystallographic Data Center Software Ltd., Cambridge, UK) generated at least 10 different poses for each corrole derivative and the pose with the largest binding affinity (corresponding to the highest positive score value and more buried into the protein cavity) was considered the best pose and analyzed in terms of interaction. Table 1 summarizes the largest docking score value for all corrole derivatives, e.g., for M^pro^ the highest score was obtained to **C2** (*meso*-aryl-corrole) than for **C1** (corrole): 35.68 and 25.76 dimensionless, respectively. The insertion of fluorine atoms into the phenyl moiety directly impacted the binding capacity of C_6_H_5_-corrole as revealed by the increase in the docking score value. Additionally, the docking score for the fluorinated-corroles **C3**–**C17** (Table 1) suggested that the position of the fluorine atoms drastically impacted the score. As an example, the docking score value for the mono-substituted-fluorinated-corroles **C3**–**C5** decreased drastically for fluorine atoms in the *para* position: 35.98, 36.44, and 24.65 dimensionless for M^pro^:**C3**/**C4**/**C5**, respectively.

**Figure 2:**
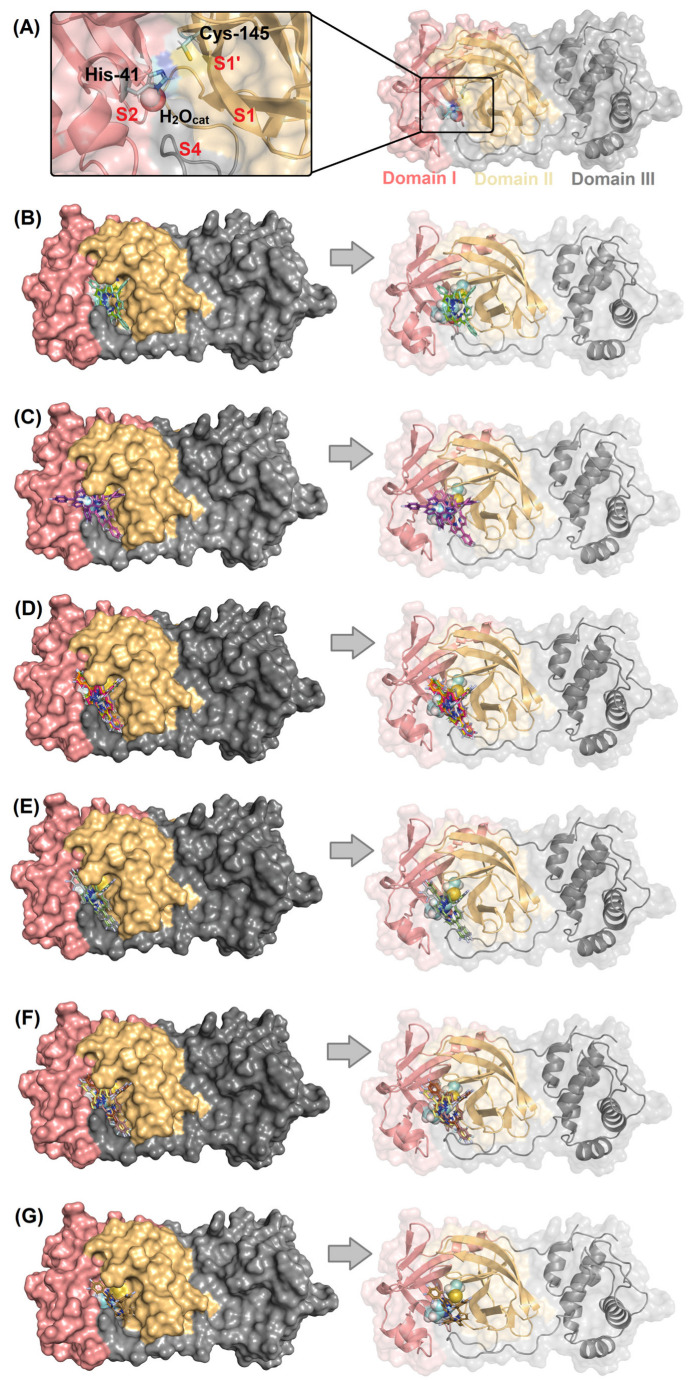
(**A**) The 3D structure for M^pro^ (PDB code: 6LU7) highlighting the catalytic Cys-His dyad (as stick in cyan) and catalytic water (H_2_O_cat_) as a sphere. Superposition of the best docking pose for the interaction between M^pro^ (as surface) with (**B**) non-fluorinated-corroles (**C1**, **C2**), (**C**) mono-fluorinated-corroles (**C3**–**C5**), (**D**) di-fluorinated-corroles (**C6**–**C11**), (**E**) tri-fluorinated-corroles (**C12**, **C13**), (**F**) tetra-fluorinated-corroles (**C14**–**C16**), and (**G**) penta-fluorinated-corroles (**C17**). For better interpretation, only the monomer and not the dimer form of M^pro^ was represented. The chemical structure and the corresponding carbon’s color of each corrole that was used in this study are in stick representation according to the 3D chemical structure of the Figure 1. Elements’ color: hydrogen, nitrogen, fluorine, and oxygen in white, dark blue, light blue, and red, respectively.

**Figure 3:**
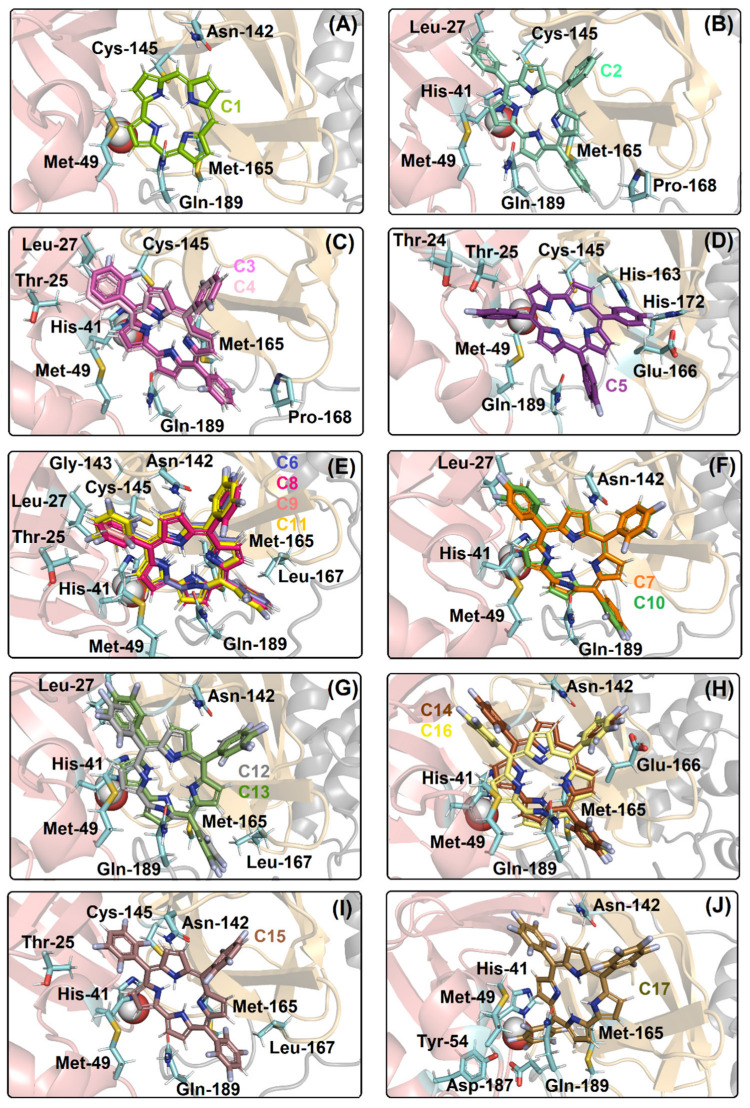
The best docking pose into the catalytic site of M^pro^ for (**A**) **C1**; (**B**) **C2**, (**C**) **C3**, **C4**; (**D**) **C5**; (**E**) **C6**, **C8**, **C9**, **C11**; (**F**) **C7**, **C10**; (**G**) **C12**, **C13**; (**H**) **C14**, **C16**; (**I**) **C15**; and (**J**) **C17**. The H_2_O_cat_ is represented as a sphere, while the selected amino acid residues that are interacting with the corrole structure are in stick representation in cyan. The M^pro^ structure is in cartoon representation, being divided into domains I, II, and III in beige, light orange, and gray, respectively. The chemical structure and the corresponding carbon’s color of each corrole that was used in this study are in stick representation according to the 3D chemical structure of the Figure 1. The elements’ color: hydrogen, nitrogen, fluorine, and oxygen in white, dark blue, light blue, and red, respectively.

Figure 2A depicts the M^pro^ enzyme and the catalytic dyad that is composed by His-41 and Cys-145 residues with the presence of a key water molecule that is important for the catalysis. Figure 2B–G show the superposition of the best docking pose for the interaction between M^pro^ with the corresponding non- (**C1**, **C2**) mono- (**C3**–**C5**), di- (**C6**–**C11**), tri- (**C12**, **C13**), tetra- (**C14**–**C16**), and penta- (**C17**) fluorinated-corroles. According to the surface representation of the M^pro^ structure, all the compounds under study are buried into the catalytic pocket assuming different binding profiles depending on the fluorinated position in C_6_H_5_-corrole structure. Table 2 summarizes the amino acid residues with the corresponding intermolecular force and distance that is involved in the interaction process M^pro^:**C1**–**C17**. In this case, van der Waals interactions was suggested as the main intermolecular force and hydrogen bonding was also detected, except for **C2**. Figure 3 depicts the 3D interaction of each corrole in the M^pro^ catalytic site, highlighting the key amino acid residues that might interact with each compound.

### 2.2. Molecular Docking Evaluation for PL^pro^ Enzyme

For the other SARS-CoV-2 protease, known as PL^pro^, Table 1 also summarizes the largest docking score value for all corrole derivatives, suggesting a higher score value for **C2** (*meso*-aryl-corrole) than **C1** (corrole) with values of 32.44 and 25.25 dimensionless, respectively. The insertion of fluorine atoms in the phenyl moiety also directly impacted the binding capacity of C_6_H_5_-corrole to PL^pro^ as revealed by the increase in the docking score value. However, differently from M^pro^, the fluorine position in **C3**–**C17** did not show any specific trend (Table 1); as an example, the docking score value for the mono-substituted-fluorinated-corroles **C3**–**C5** are quite similar: 30.41, 31.76, and 30.38 dimensionless for **C3**, **C4**, and **C5**, respectively. Nevertheless, a certain increase was noticed in the binding affinity of fluorinated-corroles to PL^pro^ with the increase of fluorine atoms in the *meso*-aryl-corrole structure, mainly from tri-fluorinated insertions, e.g., a docking score value for **C13**–**C17** is higher than 35 dimensionless.

**Figure 4:**
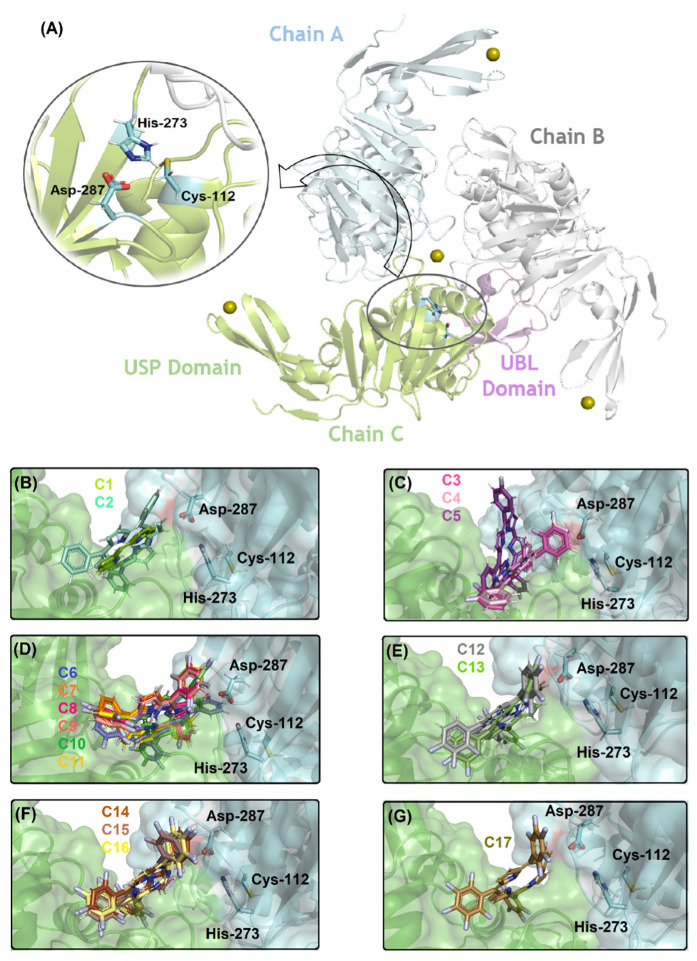
(**A**) The 3D structure for PL^pro^ (PDB code: 6W9C) highlighting the catalytic Cys-His-Asp triad (as stick in cyan). Superposition of the best docking pose for the interaction between PL^pro^ (as surface) with (**B**) non-fluorinated-corroles (**C1**, **C2**), (**C**) mono-fluorinated-corroles (**C3**–**C5**), (**D**) di-fluorinated-corroles (**C6**–**C11**), (**E**) tri-fluorinated-corroles (**C12**, **C13**), (**F**) tetra-fluorinated-corroles (**C14**–**C16**), and (**G**) penta-fluorinated-corroles (**C17**) into the catalytic triad pocket. The PL^pro^ structure is in cartoon representation divided into chains A, B, and C in blue, light gray, and green, respectively. The *N*-terminal ubiquitin-like (UBL) and *C*-terminal ubiquitin-specific (USP) domains are in violet and green (the β-sheets which interact with Zn(II) ion) into chain C, respectively. The Zn(II) ions are in sphere representation in olive, and the corresponding carbon’s color of each corrole that was used in this study is in stick representation according to the 3D chemical structure of the Figure 1. The elements’ color: hydrogen, nitrogen, fluorine, and oxygen in white, dark blue, light blue, and red, respectively.

**Figure 5:**
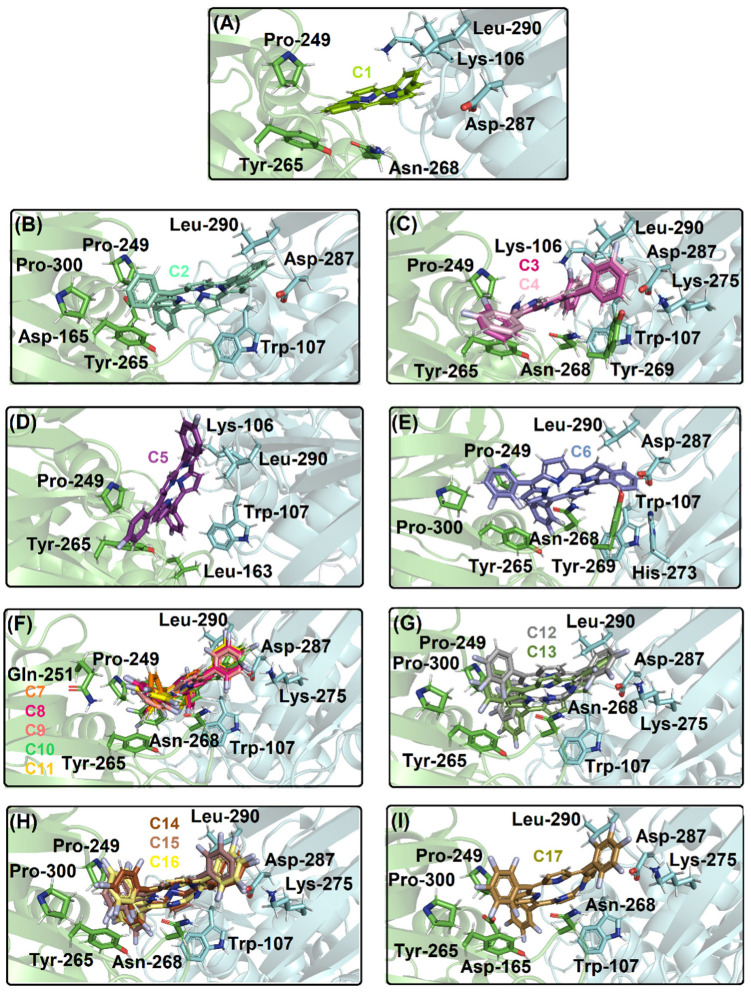
The best docking pose into the catalytic site of PL^pro^ for (**A**) **C1**; (**B**) **C2**; (**C**) **C3**, **C4**; (**D**) **C5**; (**E**) **C6**; (**F**) **C7**, **C8**, **C9**, **C10**, **C11**; (**G**) **C12**, **C13**; (**H**) **C14**, **C15**, **C16**; and (**I**) **C17**. The PL^pro^ structure is in cartoon representation divided into chains A, B, and C in blue, light gray, and green, respectively. The selected amino acid residues into chain A and C are in stick representation in blue and green, respectively. The chemical structure and the corresponding carbon’s color of each corrole that was used in this study is in stick representation according to the 3D chemical structure of the Figure 1. The elements’ color: hydrogen, nitrogen, fluorine, and oxygen in white, dark blue, light blue, and red, respectively.

Figure 4A depicts the PL^pro^ structure and the catalytic triad that is composed by Cys-112, His-273, and Asp-287 residues without the necessity of specific internal water to occur the catalysis. Figure 4B–G show the superposition of the best docking pose for the interaction between PL^pro^ with the corresponding non- (**C1**, **C2**) mono- (**C3**–**C5**), di- (**C6**–**C11**), tri- (**C12**, **C13**), tetra- (**C14**–**C16**), and penta- (**C17**) fluorinated-corroles. According to the surface representation of the protease structure, all the compounds can be buried between the catalytic pocket of chain A (in blue surface) and in part into the surface of not a catalytic pocket of chain C (in green surface) assuming a different binding profile. Curiously, for mono-fluorinated-corroles, even though the docking score value did not show a decrease in the binding affinity with fluorine at the *para* position (R_3_), from Figure 4B there is an indication that **C5** is not interacting preferentially in the catalytic site compared with **C3** and **C4**. Table 3 summarizes the amino acid residues with the corresponding intermolecular forces and distance that is involved in the interaction process with the corrole derivatives indicating van der Waals interactions as the main intermolecular force that is responsible for the interaction between PL^pro^:**C1**–**C17**, however, hydrogen bonding was also detected, except for **C1**, **C2**, and **C5**. In addition, Figure 5 depicts the 3D interaction of each corrole into PL^pro^ catalytic site highlighting the key amino acid residues that might interact with each compound.

### 2.3. 2D Quantitative Structure–Activity Relationship (2D-QSAR) Model

In attempt to correlate the docking score values that were obtained for M^pro^ and PL^pro^ with the corroles **C1**–**C17**, initially these values were correlated with the electronic effects (Hammett constants, dual parameters, Swain–Lupton inductive, and resonance parameters) and lipophilic (logP) effects. A sigmoidal correlation between the docking score values for M^pro^:**C1**–**C17** with dual electronic parameters (σ_I_ and σ_R_) with R^2^ = 0.9953 was observed. On the other hand, regarding the protease PL^pro^, a significant correlation with the investigated parameters was not observed.

Additionally, some molecular descriptors such as polarizability (POLZ), superficial tension (ST), molar volume (MV), molar refractivity (MR), and the ^13^C NMR chemical shifts of the sp^2^ carbon atoms (δ_C-1_ and δ_C-2_) adjacent to the fluorinated ring were calculated for each *meso*-aryl-corrole (**C2**–**C17**) from ACD/ChemSketch software [40] (Table 4). To understand the interaction among these compounds with M^pro^ or PL^pro^ proteases, several correlation equations were obtained by BuildQSAR 1.0 software (Universidade Federal do Espírito Santo, Vitória, Espírito Santo, Brazil) [41]. From the results that were obtained via molecular descriptors, there is the indication of only one model (M^pro^ score value and ^13^C NMR chemical shifts of the sp^2^ carbons atoms, δ_C-1_ and δ_C-2_, adjacent to the fluorinated ring, Equation (1)) with robust statistics parameters that respect the quality of the adjustment of the data in the 2D-model: the correlation coefficient (*r*^2^) and Fisher (*F*) for the statistical significance values of 0.96 and 301.92, respectively, and predictability through cross validation (*q*^2^) and the standard deviation of cross validation (*S_PRESS_*) values of 0.94 and 1.463, respectively [42,43].

M^pro^ score = 34.79 (±4.32) δ_C-1_ − 33,001.18 (±4469.79) (1)

(*n* = 15; *r*^2^ = 0.96; *s* = 127.66; *F* = 301.92; *q*^2^ = 0.94; *S_PRESS_* = 1.463; outlier: **C15**)

where *n* is the number of data points and *s* is the standard deviation. The outliers were identified by BuildQSAR software [41] and were then removed if: |Y_obs_ − Y_calc_|≥ 2 × standard deviation.

## 3. Discussion

The molecular docking technique has become one of the most used methods for determining the drug targets, offering an atomic-level explanation on the binding capacity for screening potential compounds for future in vitro assays [44,45,46]. This approach has been widely explored for repurposing clinically approved drugs, natural products, or novel synthetic compounds as potential candidates to inhibit SARS-CoV-2 replication via the inhibition of viral proteases [10,11,12,13,14,15,16,44]. Thus, in silico calculations via molecular docking was applied to suggest the interaction mechanism of 17 potential synthetic corrole derivatives (C_6_H_5_-corrole) into both M^pro^ and PL^pro^ proteins of SARS-CoV-2. Additionally, the fluorine effect on C_6_H_5_-corrole structure was also evaluated in terms of binding affinity and 2D-QSAR correlations. The tested compounds will pave the way for the development/design of drugs to inhibit SARS-CoV-2.

The M^pro^ structure is a dimer, and each monomer is divided into three different domains: domains I (8–100 residues), II (101–183 residues), and III (200–303 residues) presenting the amino acid residues His-41 and Cys-145 (catalytic dyad) in the domains I and II, respectively (catalytic site is not close to the dimer interface). The substrate-binding site is positioned inside a cleft (pockets S1, S1′, S2, and S4) between domains I and II [44,47]. It has been reported that the imidazole from His-41 residue makes a strong hydrogen bond with a water molecule (previously namely as H_2_O_cat_), suggesting that this water may play a role as a third catalytic residue, completing the noncanonical catalytic triad in M^pro^ [48,49,50] (Figure 2A). Thus, all molecular docking calculations were carried out considering the presence of H_2_O_cat_ into the catalytic pocket in the position corresponding to the crystallographic structure of M^pro^ [51].

In this case, the addition of phenyl rings in the *meso* positions (corresponding to the carbons 5, 10, and 15) of the corrole core significantly increased the docking score value, from 25.76 to 35.68 dimensionless for **C1** and **C2**, respectively, indicating that the increase of the ligand area through phenyl moieties did not negatively impact the binding capacity (theoretical area of 306.19 and 544.98 Å^2^ for **C1** and **C2**, respectively) being able to be accommodated inside the protein pocket, without a hindrance, and increase the connecting points. The insertion and position of fluorine atoms into the phenyl moiety (**C3**–**C17**) directly impacted the binding capacity of C_6_H_5_-corrole, as an example the docking score value for the mono-substituted-fluorinated-corroles **C3**–**C5** decreased drastically for the fluorine atom in the *para* position, suggesting a possible electrostatic dependence. The total analysis of the docking score value for all fluorinated-corroles to M^pro^ clearly suggested that independently of the number of fluorine atoms in the phenyl ring, at least one fluorine atom in the *para* position decreased the docking score value, showing a quite similar value compared with **C1** (Table 1), reinforcing the possible electrostatic dependence. On the other hand, all the fluorinated-corroles without a fluorine atom in the *para* position increased the binding affinity of the corroles to M^pro^ (Table 1). In this sense, when analyzing the same group corresponding to the number of fluorine atoms in the phenyl moiety, the ligands presented different poses (there is not a clear superposition), which is mainly impacted with fluorine atoms in the *para* position.

Van der Waals interactions and hydrogen bonding corroborate with the binding capacity of M^pro^:**C1**–**C17**, however, hydrogen bonding was not detected in **C2**. There is a connect point dependence for the ranking of the binding affinity of the corroles to M^pro^; as an example, the corrole **C1** which presented a lower docking score value than **C2**, showed a total of five connected points (Met-49, Asn-142, Cys-145, Met-165, and Gln-189 residues), while **C2** showed seven connected points (Leu-27, His-41, Met-49, Cys-145, Met-165, Pro-168, and Gln-189 residues). The same trend was also observed for all the fluorinated compounds comparing di- (**C6**–**C11**), tri- (**C12**, **C13**), or tetra- (**C14**–**C16**) fluorinated-corroles with the fluorine atoms in the *para* position with those in the *ortho* and/or *meta* position into the phenyl moieties. As an example, the di-fluorinated-corroles with fluorine atoms in the *para* position (**C7** and **C10**) presented five connected points (Leu-27, His-41, Met-49, Asn-142, Gln-189 residues), while the di-fluorinated-corroles with fluorine atoms in the *ortho*/*meta* positions (**C6**, **C8**, **C9**, and **C11**) presented ten connected points (Thr-25, Leu-27, His-41, Met-49, Asn-142, Gly-143, Cys-145, Met-165, Leu-167, and Gln-189 residues). Differently for the cited fluorinated-corroles, the mono-fluorinated-corroles did not show a connected point dependence, but there is a significant difference in the docking pose compared to compounds **C3**/**C4** with **C5**, suggesting that the binding capacity of the studied mono-fluorinated-corroles is closely dependent on the arrangement (buried or not) inside the protein cavity.

The molecular docking calculations also suggested that the good binding affinity of **C2** and *ortho*/*meta*-fluorinated-corroles is also dependent on the capacity to interact with the two amino acid residues that are responsible for the catalytic dyad (His-41 and Cys-145), while **C1** and *para*-fluorinated-corroles presented an interaction with just one amino acid residue of the catalytic dyad, e.g., the compounds **C3** and **C4** interacted with His-41 and Cys-145 residues via van der Waals forces within a distance of 2.70 and 3.00 Å, respectively, while the compound **C5** only interacted with Cys-145 residue within a distance of 3.70 Å (Table 2).

Differently from the M^pro^ enzyme, the PL^pro^ is a trimer that is composed of chains A, B, and C whose activity is related to the triad catalytic amino acid residues that are composed of Cys-112, His-273, and Asp-287, without the contribution of a H_2_O_cat_ [52]. Molecular docking results suggested that the presence of phenyl moieties in the corrole structure positively impacted the binding capacity of this corrole, improving the docking score value of seven punctuation, probably not due to the increase of connecting points but due to the proximity of the ligand structure with the amino acid residues of the protease (Table 3). The fluorine atoms in the phenyl moieties also impacted the binding capacity of *meso*-aryl-corroles to PL^pro^. In this case, opposing the results that were obtained for M^pro^ compared to the docking score values for fluorinated-corroles to PL^pro^, there is not any trend based on the fluorine position, specifically in the *para* position (R_3_). However, a feasible trend can be noticed based on the insertion at least tri-fluorine atoms into phenyl moieties that increased the docking score value and fitted better into the PL^pro^ catalytic pocket.

For PL^pro^, molecular docking calculations suggested van der Waals as the main intermolecular forces that are responsible for the complex stabilization, however, hydrogen bonding was also detected, except for **C1**, **C2**, and **C5**. In the present model for PL^pro^:**C1**–**C17** was not detected with a specific trend based on connecting points and hydrogen bonding as previously described for M^pro^.

The calculated ligand affinity to its target significantly depends on the charge states of the residues in the binding pocket and even the ligand [53]. Therefore, it is important to highlight that the GOLD 2020.2 software that was used in this work for molecular docking added hydrogen atoms to the proteases following tautomeric states and ionization data at a physiological pH (pH = 7.4). Since the evaluated corroles theoretically interact with the catalytic site of the proteases which harbors the catalytic amino acid residues His-41 and Cys-145 in M^pro^ and Cys-112, His-273, and Asp-287 in PL^pro^, from a mechanistic point of view, the amino acid residue Cys should be a nucleophile while His is similar to a general acid that there is the assistance of the negatively charged Asp in the case of PL^pro^ [54]. Thus, in this work, Cys and His are in the neutral form which are less likely to be stable in vitro or in vivo from a mechanistic way (Cys should have a significantly downshifted pK_a_ so that it is charged, while the His should have a high pK_a_ to stay charged) [55].

The 2D-QSAR models obtained a correlation of the docking score values for M^pro^, while for PL^pro^ there is not a clear correlation. In fact, no 2D-QSAR model was obtained using MR, MV, and POLZ descriptors and they were not useful. However, it was shown that δ_C-1_ was present as a descriptor in the 2D-model, because without it there would be no proposition of the model. For M^pro^:**C1**–**C17,** a sigmoidal correlation between the docking score values and dual electronic parameters (σ_I_ and σ_R_) was obtained, indicating two separated regions of higher and lower affinity. In this case the lower affinity and score values were observed for the corroles with a fluorine atom in the *para* position (R_3_). From the correlation between the docking score value of M^pro^:**C2**–**C17** and ^13^C NMR chemical shifts of the sp^2^ carbons atoms (δ_C-1_ and δ_C-2_) that were adjacent to the fluorinated ring a favored mathematical model by downfield of the δ_C-1_ was observed, reinforcing that the interaction between M^pro^ and C_6_H_5_-corroles might be increasing due to the decrease in the electronic density of the C_6_H_5_-corrole core. Since hydrogen bonding (HB) is strongly affected by electronic density, probably these effects can be due to HB interactions. Moreover, the 2D-model indicated an independent variable that contributes more to the model, probably due to the van der Waals interactions which were previously described in the molecular docking as the main intermolecular forces for the interaction between the studied corroles with M^pro^. The obtained 2D-QSAR model might be improved by the incorporation of experimental data, even from the literature using the same organic class that was assayed in this work, however, there are few reports on the application of tetrapyrrolic macrocycles (porphyrinoid molecules) to inhibit SARS-CoV-2 and these reports only focus on spike protein interactions which is not the target of this work [56,57,58].

## 4. Materials and Methods

### 4.1. In Silico Calculations Procedure

The three-dimensional structure of the SARS-CoV-2 proteases PL^pro^ and M^pro^ was obtained from the Protein Data Bank with access code 6W9C and 6LU7, respectively [51,52]. Spartan’18 software (Wavefunction, Inc., Irvine, CA, USA) [59] was used to build the three-dimensional structure of the corroles **C1**–**C17**. The same software optimized the chemical structure of the ligands by Density Functional Theory (DFT) approximation. GOLD 2020.2 software (Cambridge Crystallographic Data Center Software Ltd., Cambridge, UK) [60] was used to add hydrogen atoms to the structure of the proteases according to ionization at pH 7.4 and tautomeric states were inferred by the software. The same software was used for molecular docking calculations defining an 8 Å radius around the active binding site of each enzyme. The scoring function ASP was used in the docking calculations due to the lowest root mean square deviation (RMSD) that was obtained via redocking studies. PyMOL Delano Scientific LLC software (DeLano Scientific LLC: San Carlos, CA, USA) [61] was used to identify the main amino acid residues that interact with the ligands and to build the final representation.

### 4.2. The 2D-QSAR Procedure

Molecular descriptors for each *meso*-aryl-corrole (**C2**–**C17**) were drawn by ACD/ChemSketch 4.0 software (ACDLabs software package, version 12.0, Toronto, ON, Canada) [40]. The physicochemical properties were predicted by this software using additive atomic or group increments (depending on the bonds, e.g., single, double, and aromatic of an atom and on its neighboring atoms). The ACD/ChemSketch algorithm is composed of basic and derived macroscopic properties using a large experimental database relating structure to density, refractive index, and surface tension [62].

The following parameters were obtained: polarizability (POLZ), superficial tension (ST), molar volume (MV), molar refractivity (MR), and the ^13^C nuclear magnetic resonance (NMR) chemical shifts of the sp^2^ carbons atoms (δ_C-1_ and δ_C-2_) adjacent to the phenyl moiety containing fluorine atoms. The 2D-QSAR data was carried out by multiple regression analyses. All these correlations were performed by the BuildQSAR 1.0 software (Universidade Federal do Espírito Santo, Vitória, Espírito Santo, Brazil) [41]. The best descriptors were obtained by genetic algorithm, and the 2D-QSAR model was processed in terms of the highest correlation coefficient or *F*-test equations, and the lowest standard deviation equations following literature [40]. The correlation equations were obtained along with the statistical parameters, as *n* is the number of data points, *r* is the correlation coefficient, *s* is the standard deviation, *q*^2^ is the cross validation, *S_PRESS_* is the standard deviation of the cross validation (*q^2^* and *S_PRESS_* are the cross-correlation), and *F* is Fisher value measure for the statistical significance. In the equations of this work, the numbers in parentheses represent the 95% confidence intervals of the coefficients [40,41,43,63]. Also, the correlations with the score values that were obtained from molecular docking for M^pro^ and PL^pro^ with electronic parameters [64] (Hammett constants, dual parameters, and Swain–Lupton inductive and resonance parameters) and lipophilic parameter (logP, [65]) were performed using the Origin 6.0 software (OriginLab Corporation, Northampton, MA, USA).

## 5. Conclusions

Molecular docking calculations indicated that the addition of phenyl rings in the *meso* positions of the corrole core positively impacted the interaction with both proteases of SARS-CoV-2, increasing the docking score value. The insertion and position of fluorine atoms into the phenyl moieties directly impacted the binding capacity of C_6_H_5_-corrole. In the case for M^pro^, independently of the number of fluorine atoms in the phenyl rings, at least one fluorine atom in the *para* position decreases the docking score value, negatively impacting the interaction with this protease. Moreover, the 2D-QSAR model for M^pro^:**C1**–**C17** showed a sigmoidal correlation between the docking score values and dual electronic parameters (σ_I_ and σ_R_), indicating two separated regions of higher and lower affinity, as well as was obtained one mathematical model correlating docking score values (M^pro^:**C2**–**C17**) and the ^13^C NMR chemical shifts of the sp^2^ carbons atoms (δ_C-1_ and δ_C-2_) adjacent to the fluorinated ring, corroborating with the molecular docking trend. On the other hand, the molecular docking results for PL^pro^ indicated that the binding capacity into the PL^pro^ catalytic pocket increased with the insertion of tri- or more fluorine-atoms into phenyl moieties. Overall, corrole and *meso*-aryl-corrole might interact indiscriminately in the M^pro^ or PL^pro^ catalytic site, while the fluorinated-corroles which do not present *para*-fluorine atoms (**C2**, **C3**, **C4**, **C6**, **C8**, **C9**, **C15**, and **C11**) might interact preferentially in the M^pro^ catalytic site. Despite the results being only based on a molecular modeling approach (one initial step to identify potential drugs), we consider the evaluated fluorinated corroles as potential compounds for future experimental assays in inhibiting SARS-CoV-2 replication by targeting viral proteases, mainly M^pro^.

## Figures and Tables

**Table 1:** The highest molecular docking score value (dimensionless) for the interaction between M^pro^/PL^pro^ and each corrole under study (**C1**–**C17**).

Compound	M^pro^	PL^pro^
**C1**	25.76	25.25
**C2**	35.68	32.44
**C3**	35.98	30.41
**C4**	36.44	31.76
**C5**	24.65	30.38
**C6**	34.98	31.29
**C7**	22.43	30.13
**C8**	36.24	31.99
**C9**	34.50	31.84
**C10**	24.92	34.69
**C11**	35.64	32.39
**C12**	25.47	31.18
**C13**	26.37	40.65
**C14**	21.76	37.95
**C15**	36.57	35.07
**C16**	24.81	38.97
**C17**	22.84	39.29

**Table 2:** Molecular docking results for the interaction between M^pro^:**C1**–**C17**.

Compound	Amino Acid Residue	Interaction Type	Distance (Å)
	Met-49	Van der Waals	1.70
	Asn-142	Van der Waals	2.90
**C1**	Cys-145	Hydrogen bonding	3.20
	Met-165	Van der Waals	3.20
	Gln-189	Van der Waals	3.60
	Leu-27	Van der Waals	2.90
	His-41	Van der Waals	3.00
	Met-49	Van der Waals	1.90
**C2**	Cys-145	Van der Waals	2.60
	Met-165	Van der Waals	2.30
	Pro-168	Van der Waals	3.00
	Gln-189	Van der Waals	2.10
	Thr-25	Van der Waals	1.20
	Leu-27	Van der Waals	2.70
	His-41	Van der Waals	2.70
**C3, C4**	Met-49	Van der Waals	2.40
	Cys-145	Van der Waals	3.00
	Met-165	Van der Waals	2.60
	Pro-168	Van der Waals	2.80
	Gln-1896	Hydrogen bonding	3.70
	Thr-24	Hydrogen bonding	2.10
	Thr-25	Van der Waals	1.50
	Met-49	Van der Waals	2.50
**C5**	Cys-145	Van der Waals	3.70
	His-163	Van der Waals	1.40
	Glu-166	Van der Waals	2.40
	His-172	Van der Waals	1.60
	Gln-189	Van der Waals	2.40
	Thr-25	Van der Waals	2.60
	Leu-27	Van der Waals	1.80
	His-41	Van der Waals	2.70
	Met-49	Van der Waals	1.80
**C6, C8, C9, C11**	Asn-142	Van der Waals	1.90
	Gly-143	Van der Waals	2.60
	Cys-145	Hydrogen bonding	2.20
	Met-165	Van der Waals	2.60
	Leu-167	Van der Waals	2.70
	Gln-189	Hydrogen bonding	2.00
	Leu-27	Van der Waals	2.10
	His-41	Van der Waals	1.50
**C7, C10**	Met-49	Van der Waals	1.70
	Asn-142	Van der Waals	3.70
	Gln-189	Hydrogen bonding	2.00
	Leu-27	Van der Waals	2.20
	His-41	Van der Waals	2.40
	Met-49	Van der Waals	1.60
**C12, C13**	Asn-142	Van der Waals	2.00
	Met-165	Van der Waals	1,20
	Leu-167	Van der Waals	2.80
	Gln-189	Hydrogen bonding	2.10
	His-41	Van der Waals	3.10
	Met-49	Van der Waals	2.50
**C14, C16**	Asn-142	Van der Waals	2.50
	Met-165	Van der Waals	1.10
	Glu-166	Van der Waals	3.40
	Gln-189	Hydrogen bonding	2.10
	Thr-25	Van der Waals	2.80
	His-41	Van der Waals	2.90
	Met-49	Van der Waals	2.30
**C15**	Asn-142	Van der Waals	3.70
	Cys-145	Hydrogen bonding	2.30
	Met-165	Van der Waals	2.70
	Leu-167	Van der Waals	2.20
	Glu-189	Van der Waals	3.00
	His-41	Van der Waals	2.40
	Met-49	Van der Waals	2.70
	Tyr-54	Van der Waals	1.70
**C17**	Asn-142	Van der Waals	2.40
	Met-165	Van der Waals	1.20
	Asp-187	Van der Waals	1.30
	Gln-189	Hydrogen bonding	2.00
	H_2_O_cat_	Van der Waals	2.50

**Table 3:** Molecular docking results for the interaction between PL^pro^:**C1**–**C17**.

Compound	Amino Acid Residue/Chain	Interaction Type	Distance (Å)
	Lys-106/Chain A	Van der Waals	3.40
	Asp-287/Chain A	Van der Waals	3.10
**C1**	Leu-290/Chain A	Van der Waals	3.20
	Pro-249/Chain C	Van der Waals	1.80
	Asn-258/Chain C	Van der Waals	3.70
	Tyr-265/Chain C	Van der Waals	1.70
	Trp-107/Chain A	Van der Waals	1.80
	Asp-287/Chain A	Van der Waals	3.50
**C2**	Leu-290/Chain A	Van der Waals	0.60
	Pro-249/Chain C	Van der Waals	3.20
	Tyr-265/Chain C	Van der Waals	3.20
	Pro-300/Chain C	Van der Waals	1.80
	Lys-106/Chain A	Van der Waals	3.00
	Trp-107/Chain A	Van der Waals	1.80
	Lys-278/Chain A	Van der Waals	2.00
**C3, C4**	Asp-287/Chain A	Van der Waals	3.70
	Leu-290/Chain A	Van der Waals	3.20
	Pro-249/Chain C	Van der Waals	1.70
	Tyr-265/Chain C	Van der Waals	2.30
	Asn–268/Chain C	Hydrogen bonding	2.10
	Tyr-269/Chain C	Van der Waals	3.50
	Lys-106/Chain A	Van der Waals	3.10
	Trp-107/Chain A	Van der Waals	2.10
**C5**	Leu-290/Chain A	Van der Waals	2.60
	Leu-163/Chain C	Van der Waals	3.30
	Pro-249/Chain C	Van der Waals	2.50
	Tyr-265/Chain C	Van der Waals	3.00
	Trp-107/Chain A	Van der Waals	2.50
	Asp-287/Chain A	Van der Waals	2.90
	Leu-290/Chain A	Van der Waals	2.80
**C6**	Pro-249/Chain C	Van der Waals	1.70
	Tyr-265/Chain C	Van der Waals	2.90
	Asn-268/Chain C	Hydrogen bonding	2.90
	Tyr-269/Chain C	Van der Waals	2.20
	Pro-300/Chain C	Van der Waals	2.80
	Trp-107/Chain A	Van der Waals	3.70
	Lys-275/Chain A	Hydrogen bonding	1.10
	Asp-287/Chain A	Van der Waals	2.20
**C7, C8, C9, C10, C11**	Leu-290/Chain A	Van der Waals	1.20
	Pro-249/Chain C	Van der Waals	1.30
	Gln-251/Chain C	Hydrogen bonding	3.30
	Tyr-265/Chain C	Van der Waals	2.60
	Asn-268/Chain C	Hydrogen bonding	2.90
	Trp-107/Chain A	Van der Waals	1.90
	Lys-275/Chain A	Hydrogen bonding	2.50
	Asp-287/Chain A	Van der Waals	3.10
**C12, C13**	Leu-290/Chain A	Van der Waals	2.00
	Pro-249/Chain C	Van der Waals	1.70
	Tyr-265/Chain C	Van der Waals	3.20
	Asn-268/Chain C	Hydrogen bonding	3.30
	Pro-300/Chain C	Van der Waals	2.90
	Trp-107/Chain A	Van der Waals	1.90
	Lys-275/Chain A	Hydrogen bonding	1.70
	Asp-287/Chain A	Van der Waals	2.30
**C14, C15, C16**	Leu-290/Chain A	Van der Waals	1.40
	Pro-249/Chain C	Van der Waals	1.60
	Tyr-265/Chain C	Van der Waals	3.30
	Asn-268/Chain C	Hydrogen bonding	3.70
	Pro-300/Chain C	Van der Waals	3.40
	Trp-107/Chain A	Van der Waals	3.10
	Lys-275/Chain A	Hydrogen bonding	2.60
	Asp-287/Chain A	Van der Waals	3.40
	Leu-290/Chain A	Van der Waals	1.20
**C17**	Asp-165/Chain C	Van der Waals	1.90
	Pro-249/Chain C	Van der Waals	1.50
	Tyr-265/Chain C	Van der Waals	2.90
	Asn-268/Chain C	Hydrogen bonding	3.70
	Pro-300/Chain C	Van der Waals	2.70

**Table 4:**
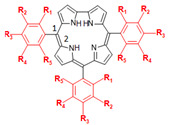
The descriptor values of molar refractivity (MR), molecular volume (MV), polarizability (POLZ), superficial tension (ST), and ^13^C nuclear magnetic resonance (NMR) shift chemical (δ_C1_ and δ_C2_) for each corrole (**C1**–**C17**).

Compound	MR	MV	POLZ	ST	δ_C1_	δ_C2_
**C1**	90.28	214.0	35.8	78.2	83.0	136.3
**C2**	164.0	409.8	65.0	63.4	105.1	135.0
**C3**	164.0	422.5	65.0	60.4	105.1	139.3
**C4**	164.0	422.5	65.0	60.4	105.1	135.0
**C5**	164.0	422.5	65.0	60.4	101.8	131.5
**C6**	164.0	435.1	65.0	57.7	105.1	139.3
**C7**	164.0	435.1	65.0	57.7	101.8	135.8
**C8**	164.0	435.1	65.0	57.7	105.1	139.3
**C9**	164.0	435.1	65.0	57.7	105.1	143.6
**C10**	164.0	435.1	65.0	57.7	101.8	131.5
**C11**	164.0	435.1	65.0	57.7	105.1	135.0
**C12**	164.0	447.7	65.0	55.2	101.8	135.8
**C13**	164.0	447.7	65.0	55.2	101.8	131.5
**C14**	164.0	460.3	65.0	53.0	101.8	135.8
**C15**	164.0	460.3	65.0	53.0	101.1	143.6
**C16**	164.0	460.3	65.0	53.0	101.8	135.8
**C17**	164.0	473.0	65.0	51.0	101.8	140.1

## Data Availability

The data presented in this study are all available in the article. Raw data are available from the authors upon request.

## Abbreviations

Chymotrypsin-like cysteine protease (3CL^pro^), main protease (M^pro^), papain-like protease (PL^pro^), severe acute respiratory syndrome coronavirus 2 (SARS-CoV-2), Middle East respiratory syndrome (MERS), 2019 coronavirus disease (COVID-19), two-dimensional quantitative structure–activity relationship (2D-QSAR), World Health Organization (WHO), ribonucleic acid (RNA), U.S. Food and Drug Administration (FDA), variants of concern (VoC), open reading frame (ORF), polarizability (POLZ), superficial tension (ST), lipophilicity (logP), molar volume (MV), molar refractivity (MR), nuclear magnetic resonance (NMR), correlation coefficient (*r*^2^), Fisher (*F*), cross validated (*q*^2^), and standard deviation of cross validation (*S_PRESS_*).
